# IMQ Induced K14-VEGF Mouse: A Stable and Long-Term Mouse Model of Psoriasis-Like Inflammation

**DOI:** 10.1371/journal.pone.0145498

**Published:** 2015-12-21

**Authors:** Xuguo Wang, Jun Sun, JinHong Hu

**Affiliations:** 1 Department of Pharmacy, Changhai Hospital, The Second Military Medical University, Shanghai, China; 2 Department of Pharmacy, General Hospital of Jinan Military Area, Jinan, Shandong province, China; University of Alabama at Birmingham, UNITED STATES

## Abstract

An imiquimod (IMQ) induced wild type (WT) mouse can mimic some features of psoriasis, such as thickened skin, abnormal keratinocyte-related proteins, infiltration of inflammatory cells and pro-inflammatory cytokines. This model is a prevalent model that is widely used in the study of psoriasis. However, skin inflammation decreases during the eighth day when IMQ is given to WT mice, which may result in false results when evaluating the pharmacodynamics effects of a drug. To extend the timeliness and inherit the advantages of this model, we applied IMQ to the skin of 8-week-old homozygous K14-VEGF mice to investigate whether IMQ can prolong mice ear inflammation. In our experiments, we found that, compared to the IMQ induced WT mice model, the IMQ induced K14-VEGF mice have serious skin inflammation, even on the fourteenth day. We also evaluated the stability of skin inflammation at days 8, 10, and 13, and the inflammatory situation remained stable in the skin. This research intends to improve the existing model, and we hypothesize that the IMQ induced K14-VEGF mouse will become a practical mouse model in psoriasis research.

## Introduction

Psoriasis is a common chronic inflammatory skin disorder that affects 2–3% of the population[1]. It is also a genetic-related disease, and the Genome Wide Association Study (GWAS) has identified many Psoriasis susceptibility gene loci (PSORs), including Human Leukocyte Antigen (HLA-C) (rs10484544) and interleukin-12b (IL-12b) (rs3212227)[2,3]. Environmental factors, such as drugs, stress, and streptococcal infection, can also interact with the genetic factors[4]. Our understanding of the complications of psoriasis is still very superficial; however, many researchers now believe that the IL-23/Th17 axis plays a significant role in the initiation and maintenance of the disease[5]. Moreover, inflammatory cells, such as dendritic cells (DCs), macrophages, and neutrophils, are also substantially involved in psoriasis. Psoriasis also displays features of the skin neuroendocrine system disorder[6], such as the significantly increased nerve fibres which are implicated in the inhanced itch sensation for psoriatic patients[7]. Besides, histamine changes in the psoriatic epidermis can cooperate with IL-17 to augment the production of IL-8 and granulocyte-macrophage colony-stimulating factor (GM-CSF)[8]. Serotonin, highly expressed in the epidermis and cuticular appendage[9], is associated with flares of psoriasis vulgaris [6].

Currently, research of psoriasis mainly depends on the type of mouse model. In addition to genetically engineered mice models, there are some other models including the IL-23 induced[10] and IMQ induced[11] models. The toll-like receptor 7 (TLR7) agonist IMQ is used for topical treatment of actinic keratoses, superficial basal cell carcinomas, and human papilloma virus-caused genital and perianal warts[12,13], but it was found that in the treatment of people who are prone to psoriasis, the occurrence of psoriasis was detected[14]. According to these characteristics, researchers applied IMQ to mice and obtained relevant results[11]. In recent years, as the research with this model has progressed, scientists have become increasingly aware of the importance of this model and apply IMQ to many genetically engineered mice[15,16]. Accordingly, it has become one of the most important mice models in psoriasis research.

Vascular endothelial growth factor (VEGF) is a crucial factor that mediates the angiogenesis of blood vessels and is highly expressed in the skin lesions of psoriasis. VEGF induces microvascular alterations in the dermal papillae, which facilitates the development and persistence of the psoriatic lesions. Moreover, the increased vasculature and permeability provides nutrition to the hyperproliferating keratinocytes and promotes the migration of inflammatory cells[17,18]. K14-VEGF mice can develop similar phenotypes with the psoriatic lesions, and histologically, they show dermal blood vessel hyperplasia and abnormal keratinocyte proliferation, which causes skin thickening and massive infiltration of inflammatory cells[19].

K14-VEGF mice older than 6 months can develop psoriasis. We speculate that the pathologic changes in the skin of this type of mouse can favor the occurrence of psoriasis-like lesions with age. Additionally, short term inflammation in the skin of IMQ induced WT mice presents particular limitations. Thus, we applied IMQ to the skin of 8-week-old K14-VEGF mice to determine if we can extend the inflammation causing effect without influencing the existing advantages.

## Materials and Methods

### Mice and Ethics Statement

The K14-VEGF mice are produced commercially by Cyagen Biosciences Inc. (Guangzhou, China). A murine cDNA coding for the VEGF-A164 was ligated into a cassette containing the human K14 promoter. The resulting construct was then injected into FVB/N zygotes. The hemizygous pups were intercrossed to establish homozygotes as previously described[20]. In this study, homozygous K14-VEGF mice were used. FVB/N JCL mice, which were included as a control group, were purchased from the experimental animal center of the Second Military Medical University and were maintained in specific pathogen-free conditions. Animal experiments in this study were approved by the experimental animal ethics committee of the Second Military Medical University.

### Chemicals

The chemicals used in this study included 5% imiquimod propionate cream (Sichuan Med-Shine Pharmaceutical CO., LTD, China) and anti-IL-17A, IL-23, CCR6, CD11c antibodies (Abs), which were all purchased from Abcam (Cambridge, UK). Water was distilled and purified using a Milli-Q Water Purification System (Millipore, USA).

#### IMQ treatment

Imiquimod cream was applied to the inside ear of 8-week-old mice, and Vaseline was applied to the control group; the amount applied for each substance was 5 mg for each ear per day.

### Measurement of ear thickness, epidermal proliferation and scoring of ear inflammation

Ear thickness was measured using a micrometer (Guanglu Digital Caliper Manufacturer Co., Ltd, China) before the application of IMQ and Vaseline each day. Ear thickness was measured in duplicate and averaged to indicate the severity of the ear inflammation. Visiopharm software was used to determin the mean epidermal thickness (Visiopharm, Hoersholm, Denmark).

To score the inflammatory severity of the treated ear, a modified Psoriasis Area and Severity Index (PASI) score was calculated; the factors in the measure consist of erythema, thickening and scaling. Each factor was graded independently on a scale from 0–4 (0, none and 4, very marked). The cumulative score can range from 0–12.

### Real-time PCR

The mice ear samples (50 mg) were prepared for the isolation of total RNA using the Maxwell^®^ RSC simply RNA kits (promega, USA). The Promega kit (GoScript™ Reverse Transcription System) was used for the synthesis of the complementary DNA. Then, 1 ug of cDNA was used in the RT-qPCR Systems (promega, USA) on white 96-well plates. Real-time PCR was performed on a Roche Lightcycler 480II. The conditions were 95°C for 15 s and 60°C for 1 min, and a single fluorescence measurement was performed.

The sequences of the real-time PCR primers were as follows: IL-17A forward GGACTCTCCACCGCAATGA reverse TCAGGCTCCCTCTTCAGGAC. IL-23 forward CCCGTATCCAGTGTGAAGATG reverse GGGCTATCAGGGAGTAGAGCA. TNF-α forward TCTCATGCACCACCATCAAG reverse GAGGCAACCTGACCACTCTC. IFN-γ forward CTGCTGATGGGAGGAGATGT reverse TTTGTCATTCGGGTGTAGTCA. IL-1β forward CCCTGCAGCTGGAGAGTGTGGA reverse TGTGCTCTGCTTGTGAGGTGCTG. IL-6 forward ACTTCCATCCAGTTGCCTTC reverse ATTTCCACGATTTCCCAGAG. CXCL-9 forward TGTGGAGTTCGAGGAACCCT reverse AGTCCGGATCTAGGCAGGTT. CXCL-10 forward CCACGTGTTGAGATCATTGCC reverse GAGGCTCTCTGCTGTCCATC. CCL-20 forward CAGGCAGAAGCAAGCAACTAC reverse AGCTTCATCGGCCATCTGTC. GAPDH forward TGGCCTTCCGTGTTCCTAC reverse GAGTTGCTGTTGAAGTCGCA.

Primer sequences were all from 5' to 3'.

### Histology and Immunohistochemistry

The samples were processed as previously described[21]. Briefly, sections were washed in xylene to deparaffinize followed by graded alcohol series. Then paraffin sections were performed by microwaving the sections in citrate buffer. BSA were used as blocking agent for 30 min at 20°C. The sections were either stained with primary Abs from mouse CCR6 and CD11c or the corresponding isotype control Abs (IgG) for 9 hours at 4°C.

### Western-blot

The mice ear samples were harvested and cleaned in Tris-buffered saline (TBS) twice; then, they were cut into pieces and homogenized with a refiner. Western blot was performed as previously described [22]. To apply the IL-17 and IL-23 in the mouse ear, samples were incubated with polyclonal Abs against mouse IL-17 and IL-23. Bands were visualized using ECL.

### Statistical analysis

Two tailed unpaired Student’s t-tests were used to compare the two groups. For comparisons involving multiple groups, a one-way ANOVA analysis was used. Values of P are depicted as *≤0.05; **≤0.01; and ***≤0.001. In all cases, GraphPad Prism 5 software was used.

## Results

### Ear inflammation in 8-week-old K14-VEGF mice

**Fig 1:**
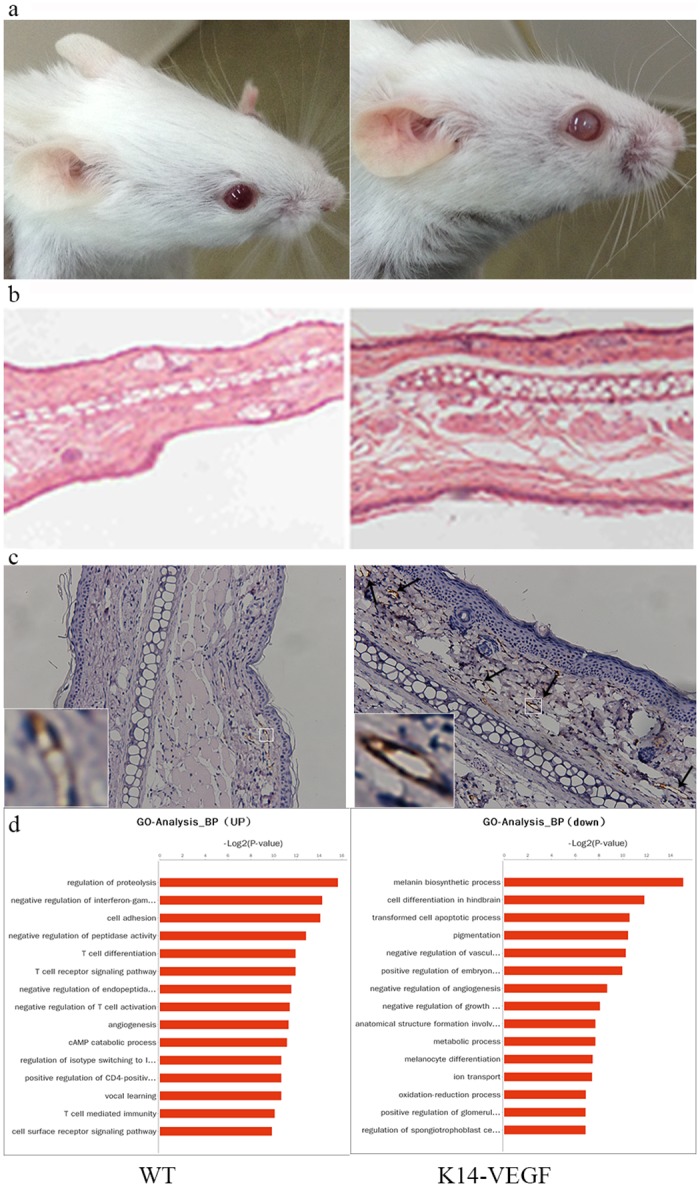
The differences between 8-week-old K14-VEGF mice and WT mice. (a) Appearance differences between WT mice (left) and K14-VEGF mice (right). (b) H&E staining of the ear of WT mice (left) and K14-VEGF mice (right). A representative of six mice from three independent experiments is shown. (c) IHC analysis of CD31-positive cells in WT mice (left) and K14-VEGF mice (right). Images are representative of five mice from three independent experiments in each group. Arrows indicate the location of the CD31^+^ cells. Scale bars, 50 μm. (d) The GO analysis of K14-VEGF mice compared with wild type mice, n = 3.

The skin of K14-VEGF mice older than 6 months can develop psoriatic-like features, including redness, hyperplasia, etc. In this experiment, we chose 8-week-old K14-VEGF mice that had skin that differed from the WT skin but did not have obvious inflammation. In appearance, the ear of the homozygous transgenic mice was redder than the wild type mice (Fig 1a). The H&E results also showed an increase in ear thickness (Fig 1b). CD31, a marker of blood and lymphatic vessels, was also analyzed. The results from the immunohistochemical analysis showed that there were some CD31-positive cells in the transgenic mice, whereas this type of cell was rare in the WT mice (Fig 1c).

To understand the comprehensive overview of the 8-week-old K14-VEGF mouse, we sent the wild type and transgenic mice with similar age to the company for a transcriptome sequencing analysis. We found that compared with the WT mice, the K14-VEGF mice had mild inflammation (shown in the GO analysis, Fig 1e).

### IMQ induces more stable ear inflammation in the skin of K14-VEGF mice than that in wild type mice

**Fig 2:**
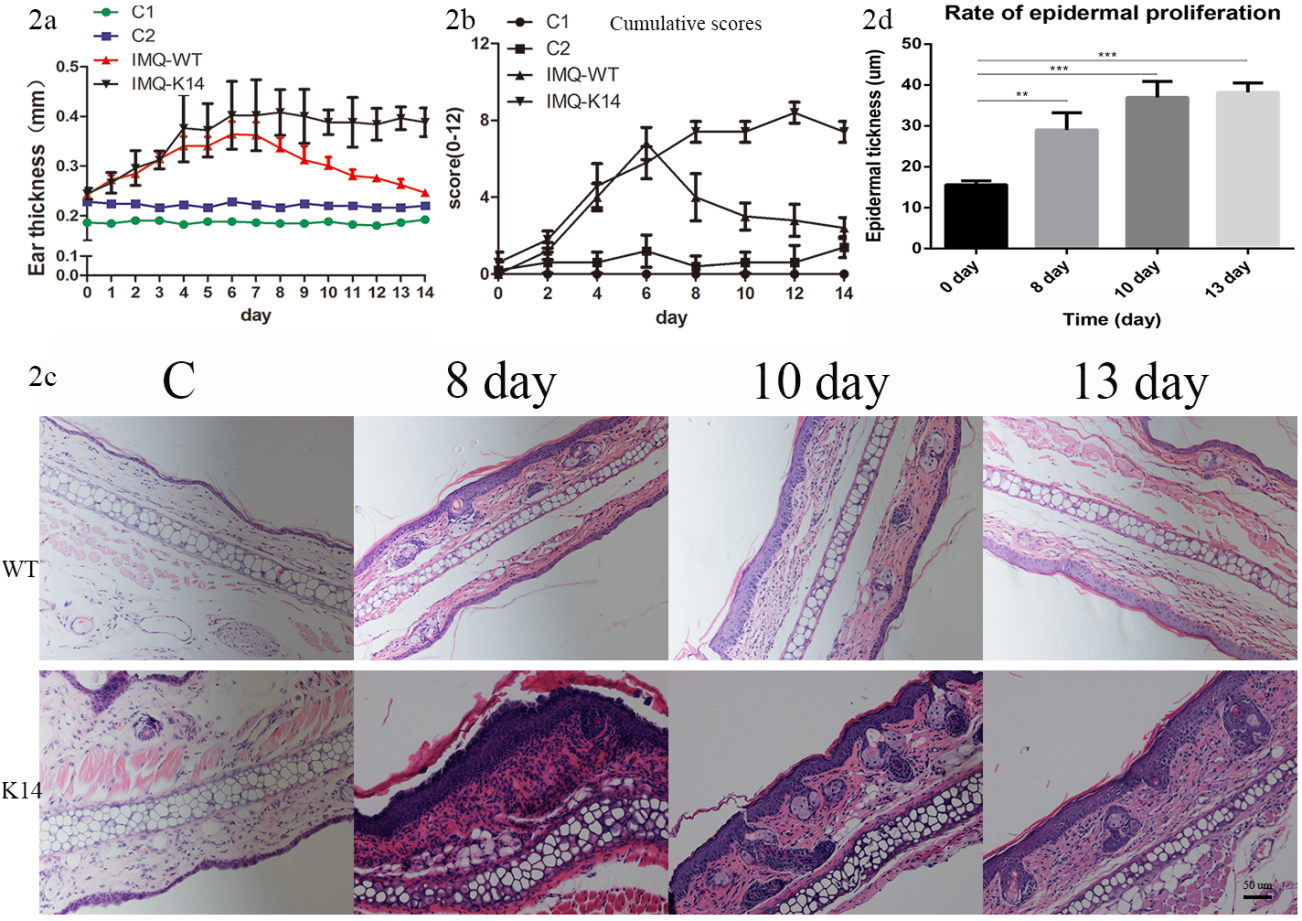
IMQ-treated K14 mice had more stable skin inflammation. (a) The thickness of the ear was measured on the days indicated (Mean±SD; n = 5 mice for each time point in each group). (b) Erythema, scaling and thickness of ear skin was assessed on the indicated days with a scale from 0 to 4. The cumulative score is presented (Mean±SD; n = 5 mice for each time point in each group). (c) H&E staining of the mouse ear skin of the treatment groups (200×). (d) Rate of epidermal proliferation in different time after appalication of IMQ.

Fig 2 shows a summary of the ear inflammation in the 4 differently treated groups. There were two control groups: C1 represents the wild type control group, and C2 represents the K14-VEGF mice control group. C2 ear samples were slightly thicker than those of C1 (Fig 2a). The maximum ear thickness of IMQ-induced wild type mice (IMQ-WT) was 0.36±0.06 mm on day 7 and decreased to 0.25±0.02 mm on day 14. The maximum ear thickness in the IMQ-treated K14-VEGF (IMQ-K14) group, was 0.40±0.07 on day 6, which was maintained almost unchanged until day 14 (0.39±0.03). The ear thickness had remained stable from day 6 to day 14 in the IMQ-K14 group, whereas the ear thickness of IMQ-WT mice increased until day 7 and began to decrease thereafter.

Disease severity was assessed using the Psoriasis Area and Severity Index score[11]. On day 2 after the IMQ application, signs of thickening, erythema, and scaling appeared in both IMQ-induced groups. Afterwards, the scores continuously increased up to day 6 and thereafter began to decrease in the IMQ-WT group. In the IMQ-K14 group, the score peaked on day 8 and remained stable until day 14. Similar to the results of ear skin thickness, the scores in the C2 group were slightly higher than those in the C1 group. The total scores are depicted in Fig 2b.

Compared with the control group, parakeratosis (retention of nuclei in the stratum corneum) and acanthosis (marked thickening of the epidermis) was observed in the IMQ-induced groups. Moreover, rete ridges, a classic characteristic of psoriasis, were also found in both IMQ-treated groups (Fig 2c and 2d).

### High level of mRNA of pro-inflammation cytokines was still induced by IMQ in the skin of K14-VEGF mice after 14 days of application

Psoriasis is caused by the disturbance of TH17 and TH1 cells in the skin lesions. Chemokine (C-X-C motif) ligand 9 (CXCL)-9/10 are chemokines of Th1 type cells, which are overexpressed in the lesions. chemokine (C-C motif) ligand 20 (CCL-20) is a chemokine of chemokine (C-C motif) receptor (CCR)6^+^ cells, and its overexpression is a typical feature in psoriatic lesions and IMQ-treated mice. Overexpressed mRNA levels were detected on the 14th day after IMQ application.

The IL-23/TH17 axis plays an important role in the pathogenesis of psoriasis. IL-23 and IL-17a were highly expressed in the IMQ-K14 mice, whereas there were no significant differences in the IMQ-WT mice compared with the relevant control mice after 14 days of IMQ application (data not shown). IL-19, a member of the IL-10 family, was highly expressed in the psoriatic lesions and plays an important role in mediating the IL-23/IL-17 axis. Its inhibition will attenuate IMQ-induced inflammation[23]. In the IMQ-K14 mice, IL-19 was still highly expressed on day 14. IL-1β and IL-6 are pluripotent pro-inflammatory cytokines, which can initiate a cascading inflammatory reaction. Additionally, IL-1β is crucial in the proliferation of TH17 cells. This type of cytokine is secreted by keratinocytes (KCs) that are induced by isostearic acid, a component in the Aldra[24]. In the IMQ-K14 mice, these cytokines were highly expressed on the 14th day.

**Fig 3:**
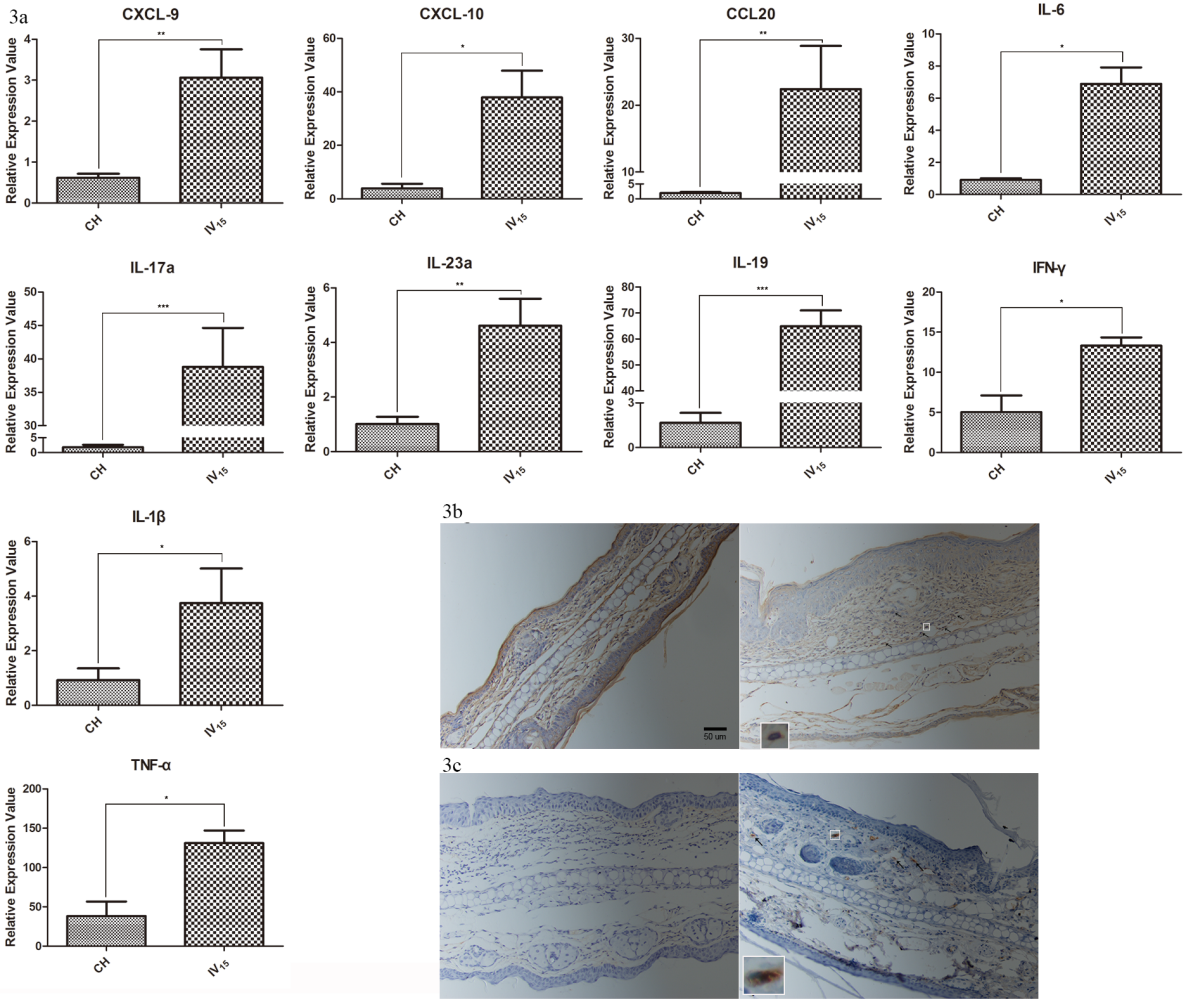
IMQ induced a longer skin inflammation period. (a) mRNA expression of some pro-inflammatory cytokines in the mice skin, n = 6. (b) IHC staining of CCR6^+^ cells in the control group and IMQ-K14 mice on the 14th day. Arrows indicate the location of the CCR6^+^ cells. (c) IHC staining of CD11c^+^ cells in the control group and IMQ-K14 mice on the 14th day. Arrows indicate the location of the CD11c^+^ cells. CH: 8-week-old K14-VEGF mice; IV_15_: IMQ-K14 14 days.

Tumor Necrosis Factor (TNF)-α and interferon (IFN)-γ have long been thought to play an essential role in psoriasis. TNF-α may be secreted by neutrophils, and KCs may be its main resource. In contrast, IFN-γ are primarily secreted by TH1 cells. In our IMQ-K14 model, both cytokines were expressed at the mRNA level at a slightly high level. All the amounts are shown in Fig 3a.

In human psoriasis, the main producer of IL-17 is the CD4^+^T cell, namely the Th17 cell. However, murine experiments have revealed that IL-17 is substantially released by γδ T cells[25,26]. CCR6 is a cell marker of both Th17 and γδ T cells, and CD11c^+^ inflammatory DCs are another significant cell type in psoriasis[27]; they were increased in the IMQ-K14 group and were barely found in the IMQ-WT mice on day 14 (Fig 3b).

### IMQ-treated K14-VEGF mice developed a stable infiltration of inflammatory cells and cytokines

**Fig 4:**
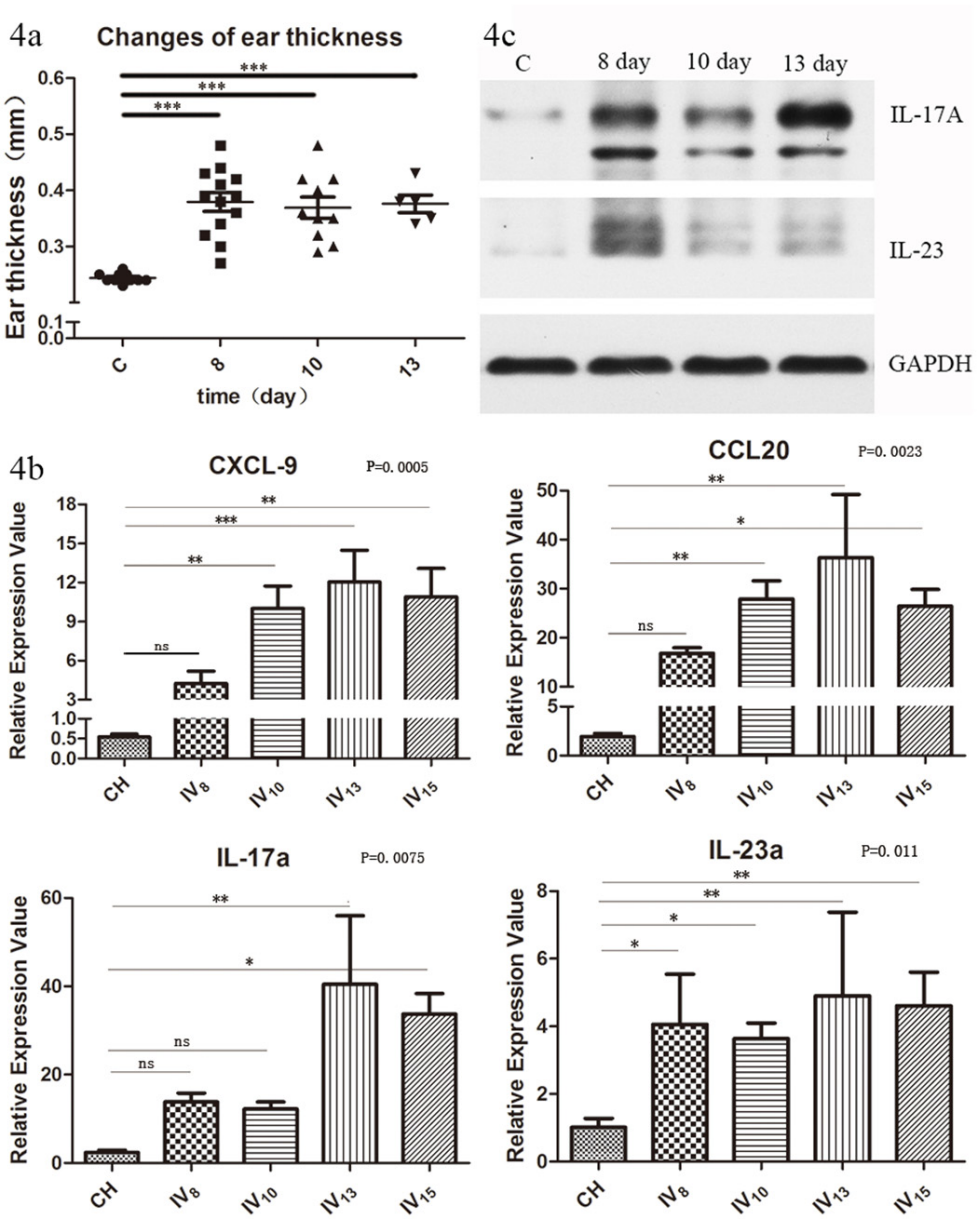
Inflammatory changes on days 8–14 in the IMQ-K14 mice. (a) Changes in ear thickness. n = 6 (b) mRNA level changes of psoriasis-related cytokines, n = 6. (c) Protein level changes of IL-17 and IL-23, n = 3.

To ensure the stability of the IMQ-K14 model, we further inspected the proinflammatory markers at another 3 time points. As the results showed that inflammation in the IMQ-WT mice began to decrease after days 6–8, we evaluated the skin inflammation changes on days 8, 10 and 13. Specifically, the mRNA levels of IL-17a, IL-23a, TNF, IFN-γ, IL-1B and IL-6 were examined (Fig 4a), and the protein levels of IL-17 and IL-23 were detected (Fig 4b).

## Discussion

Our results demonstrate that inflammation in IMQ-K14 mice is more stable and long-lasting than that in IMQ-WT mice, which may depend on the continuous expression of conventional DCs (cDCs), a main producer of IL-23[28].

As reported before, homozygous K14-VEGF transgenic mice develop a psoriasis-like phenotype at 5–6 months old, including abnormal epidermal proliferation and differentiation, epidermal microabscesses, inflammatory infiltrates and so on[19]. Therefore, we can conclude that before the visible pathological changes appears, there must be some changes occurring in the ear, such as the overexpressed VEGF-A contributing to the accumulation of IL-17A^+^ γδ T cells in mouse skin and plasmacytoid dendritic cells (pDCs) traveling to the inflammatory sites[26]. Additionally, the VEGF-responsive genes are involved in the activation of endothelial cells[29]. In our experiments, we found that 8-week-old K14-VEGF mice had a redder ear than the wild type mice, and the H&E staining revealed thicker skin in the transgenic group. The thickness was not very obvious, which may be due to the deficiency of enough inflammatory cells that can lead to the edema. The transcriptome sequencing analysis uncovered more than 300 different genes compared with the wild type. The GO analysis disclosed that the various genes mainly focused on the T cell signaling and pro-inflammation, and these changes may lead to the spontaneous symptoms in the elder K14-VEGF mice. The CoExpress Act Network revealed that the most significant distinction from the wild type mice was the mitogen-activated protein kinase kinase kinase kinase (map4k) overexpression in the K14-VEGF mice skin. Map4k1 is also known as hpk1. Its cleavage can induce cell death in T and B lymphocytes[30]. Therefore, we can conclude that the 8-week-old K14-VEGF mice are adequate for our experiments. Psoriasis is a chronic disease, so determining the factors that account for its continuous nature will help our understanding of psoriasis.

IMQ is responsible for the early and rapid accumulation of pDCs[31], neutrophil cells[11] (also see in S2 Fig) and other immune cells in the skin. These cells stimulate keratinocytes to increase cytokine production, mostly chemotactic factors such as CCL-20, and attract many adaptive immune cells to the skin[32]. The immune cells then lead to psoriasis-like dermatitis that resembles human psoriasis. It is generally believed that the psoriatic-like phenomenon in IMQ-treated mice mainly depends on the IL-23/RORγt/Th-17 axis. A more detailed mechanism is that IMQ stimulates the TLR7 on Langerin^neg^ cDCs, which leads to the production of IL-23, and this early production of IL-23 then induces the generation of IL-17 and IL-22 from innate lymphocytes (ILCs). Langerin^neg^ cDCs are the main resource of IL-23 and are, therefore, the initiating factor in the inflammatory cascade reaction[28]. Other research has also indicated that the main IL-17 secretion cells are γδ T cells and RORγt^+^ ILCs, and IL-36/IL-36R may represent the upstream signal [33]. In our experiments, the mRNA levels of IL-17, IL-22 and IL-23 were all overexpressed on day 14, and the protein levels of IL-17 and IL-23 also maintained high expression, which suggests this model can mimic the pathogenesis of psoriasis. IL-10 can extensively suppress the immune reaction. Surprisingly, the mRNA level of IL-10 was high in the IMQ-K14 mice. IL-10 may be secreted by Treg cells, and it can also be secreted by DCs in some conditions. We suspect that the large amount of IL-10 might be the product of negative immune regulation, and further experiments should be conducted to explore the impact of IL-10 in the IMQ-treated mice. Of course, there is a suspicion that the enhanced expression of immune cells observed on this study may be due to hair follicle formation (anagen) other than IMQ induction. But we believed that the random-group design in this study would deal with it well.

Although psoriasis can be found only in humans, particular animal models may be used to research the disease. An excellent model should share all of the 4 significant histopathologic traits, mimic the pathogenesis mechanism that is related to psoriasis, and respond to agents that are used to treat psoriasis. IMQ-WT mice already meet all of these criteria; however, as in the drug cure experiments, false-positive results may arise due to the decreased inflammation at days 6–8 after the application of IMQ. There are also some additional problems with the typical IMQ-WT mice; for example, there are large differences because of the inconsistency in inflammation in various mice. To improve the usability of the IMQ model, we applied IMQ to the skin of K14-VEGF mice, which developed a more stable and long-term model that is more practical. Additionally, IMQ-K14 is more appropriate for long-term studies compared with the IMQ-WT, which is a type of acute chemical-stimulated model. Differences exist between them, and the long-term model (IMQ-K14) can better mimic the progression of psoriasis.

Using IMQ-WT mice, we applied IMQ to the skin of K14-VEGF transgenic mice to obtain a more practical and reliable model. Our results strengthen the usability of the IMQ-treated animal model.

## Supporting Information

S1 Fig Clinically features of IMQ-induced K14-VEGF mouse model. (a) Scales can be found after 3 days’ IMQ application. (b) There are rete ridges presenting in the H&E staining picture.
(TIF)

S2 Fig MPO sataining of neutrophiles among different groups. MPO^+^ cells can be found in the control group seldomly (left), while the other two groups have much more MPO^+^ cells in the epidermis, and the group (4 × 24 h) have the strongest positive expressions. Scale bar = 50 um.
(TIF)

S3 Fig Ki67 staining of K14 and IMQ-induced K14 mice. There are scarce Ki67^+^ cells in the ear of K14 mice (left), and the ear basal layer of the IMQ-induced K14 mice (right) shows a high positive rate.
(TIF)

S4 Fig Ear inflamation responses with the anti-IL-17 mAb interfered. The upper left figure denotes the epidermal thickness changes and the rest figures show the mRNA changes of CCL-20, IL-6 and IL-1b.
(TIF)
