# Supplementary figures and images for: IMQ Induced K14-VEGF Mouse: A Stable and Long-Term Mouse Model of Psoriasis-Like Inflammation
Source: PLoS One. 2015 Dec 21;10(12):e0145498. doi: 10.1371/journal.pone.0145498 (PMC4687059; doi:10.1371/journal.pone.0145498)

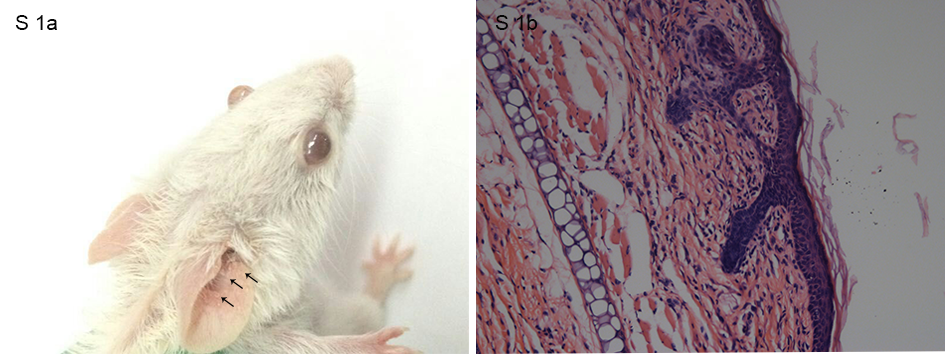

Supplement: S1 Fig — (a) Scales can be found after 3 days’ IMQ application. (b) There are rete ridges presenting in the H&E staining picture. (TIF) [file pone.0145498.s001.tif]

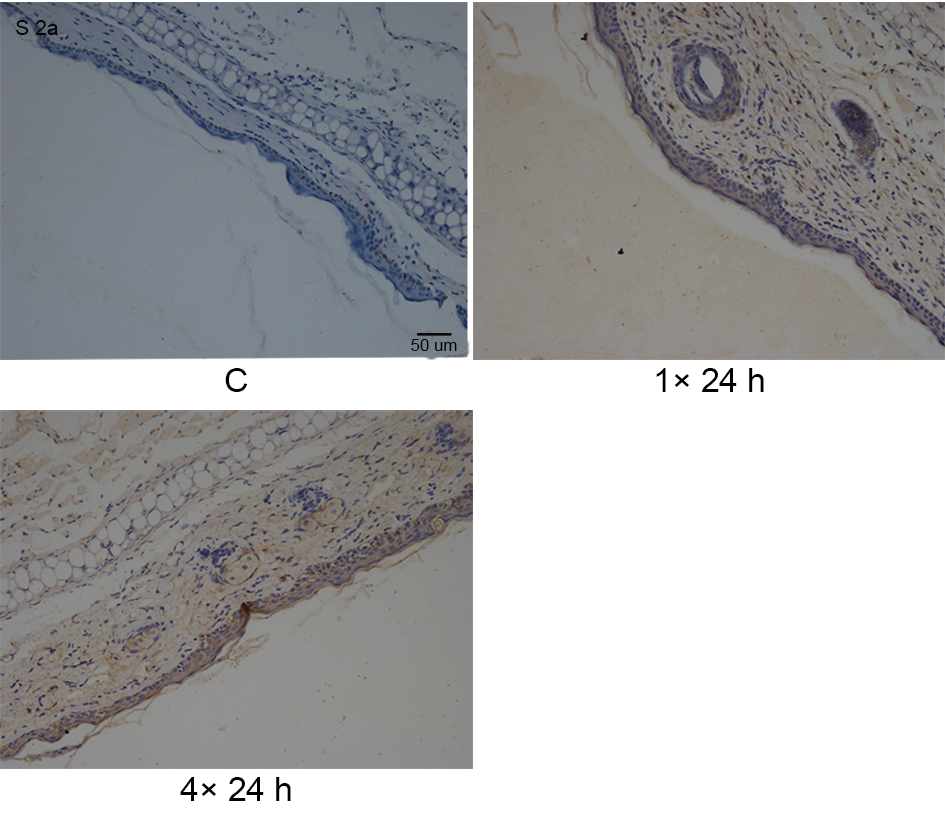

Supplement: S2 Fig — MPO+ cells can be found in the control group seldomly (left), while the other two groups have much more MPO+ cells in the epidermis, and the group (4 × 24 h) have the strongest positive expressions. Scale bar = 50 um. (TIF) [file pone.0145498.s002.tif]

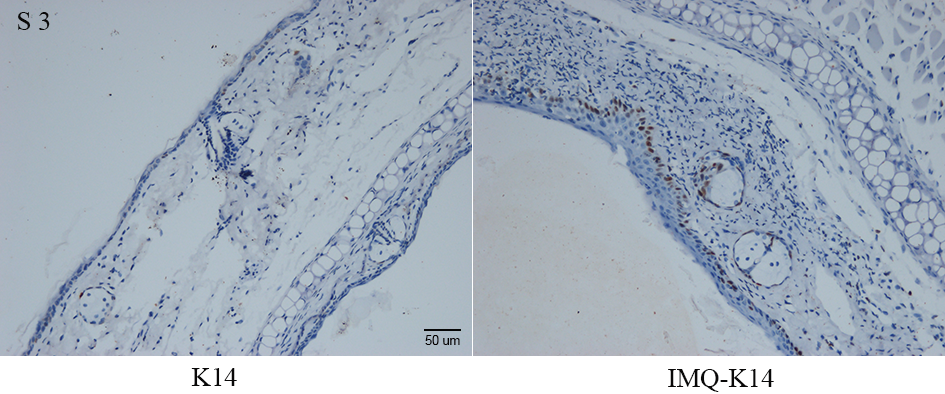

Supplement: S3 Fig — There are scarce Ki67+ cells in the ear of K14 mice (left), and the ear basal layer of the IMQ-induced K14 mice (right) shows a high positive rate. (TIF) [file pone.0145498.s003.tif]

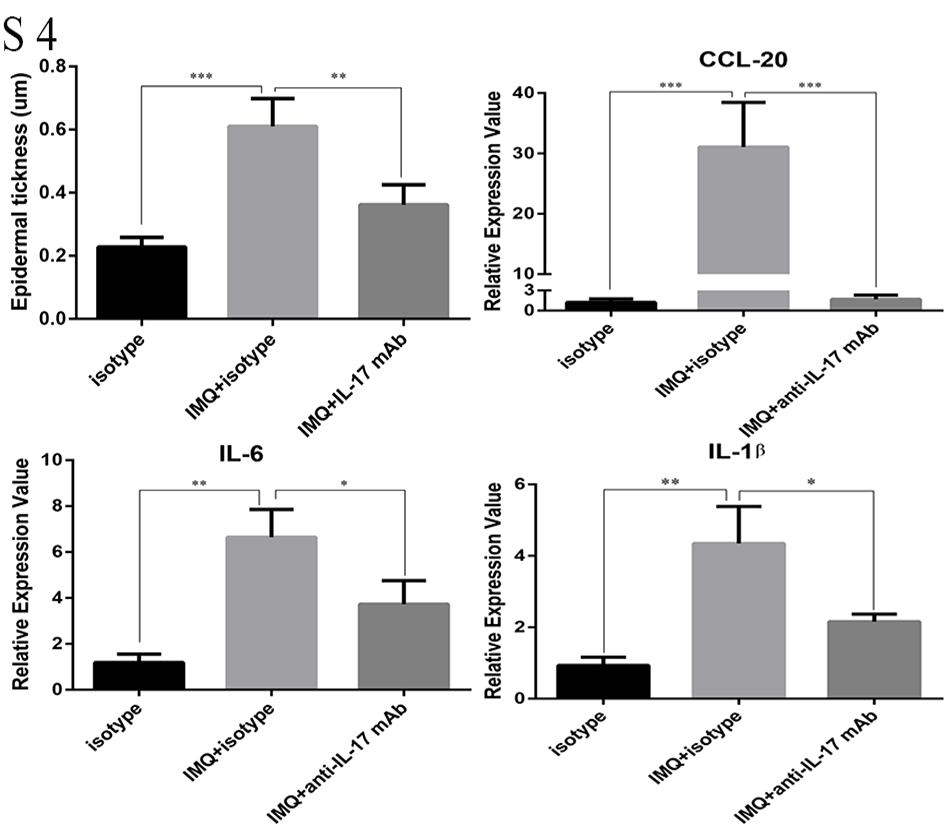

Supplement: S4 Fig — The upper left figure denotes the epidermal thickness changes and the rest figures show the mRNA changes of CCL-20, IL-6 and IL-1b. (TIF) [file pone.0145498.s004.tif]
